# The Modified Ultrasound-Assisted Method: A Study of the Correlation between Magnetic Resonance Imaging and the Ultrasound-Assisted Evaluation of the Critical Shoulder Angle

**DOI:** 10.3390/diagnostics14050486

**Published:** 2024-02-23

**Authors:** Christian T. Schamberger, Tobias Grossner, Christian Fischer, Sebastian Findeisen, Thomas Ferbert, Arnold J. Suda, Gerhard Schmidmaier, Stephan Stein

**Affiliations:** 1 Clinic for Trauma- and Reconstructive Surgery, University Clinic Heidelberg, 69120 Heidelberg, Germanychristine.gugau@med.uni-heidelberg.de (G.S.); 2 ARCUS Clinic, 75179 Pforzheim, Germany; 3 Department of Orthopaedics and Trauma Surgery, AUVA Trauma Center Salzburg, 5010 Salzburg, Austria

**Keywords:** critical shoulder angle, ultrasound, MRI, impingement

## Abstract

Background: An increased or decreased critical shoulder angle (CSA) is a known risk factor for osteoarthritis, lesions, and re-ruptures in the rotator cuff. A CSA greater than 35° correlates with degenerative rotator cuff tears, while a CSA of less than 30° correlates with osteoarthritis in the glenohumeral joint. The diagnostic gold standard for its determination is X-ray or MRI. Objectives: The primary objective of this research was to assess the viability of utilizing sonography imaging as a diagnostic tool to determine the modified critical shoulder angle (mCSA). This study aimed to investigate the feasibility and effectiveness of sonographic techniques in accurately diagnosing CSA compared to MRI. Study Design and Methods: A cohort study was carried out (level of evidence 3). The CSA (MRI) and the mCSA (ultrasound) were assessed retrospectively by two independent board-certified investigators in 109 patients with shoulder pain by MRI and musculoskeletal sonography. The CSA in the MRI dataset was determined using routine protocols and then compared to the values assessed using the modified sonography-assisted method (mCSA). Both results were analyzed with linear regression to determine a possible correlation. All investigations were performed by a DEGUM (German Society for Medical Ultrasound)-certified specialist in musculoskeletal sonography. Results: A total of 112 patients were included in this study, namely 40 female patients and 72 male patients with a mean age of 54.7 years at the time of the investigation. The mean CSA in MRI was 31.5° ± 3.899, and the mCSA in sonography was 30.1° ± 4.753. The inter- and intraobserver reliability for the CSA was factual with values of 0.993 and 0.967. The inter- and intraobserver reliability for mCSA was factual as well, with values of 0.989 and 0.948. The ANOVA analysis did not reveal a significant difference between the CSA and the mCSA values, and linear regression determined the R^2^ value to be 0.358 with *p* < 0.05. Conclusions: Diagnosing the mCSA using sonography is a safe and valid method. No statistically significant differences between the results in MRI and sonography could be seen. Although this is a retrospective, single-center study including only Caucasian mid-Europeans, and with the known limitations of ultrasound imaging, it nevertheless shows that sonography can be used as a simple, cheap, and fast technique to assess a modified CSA, which shows very good correlation with the standard CSA without losing the diagnostic quality.

## 1. Introduction

**Figure 1:**
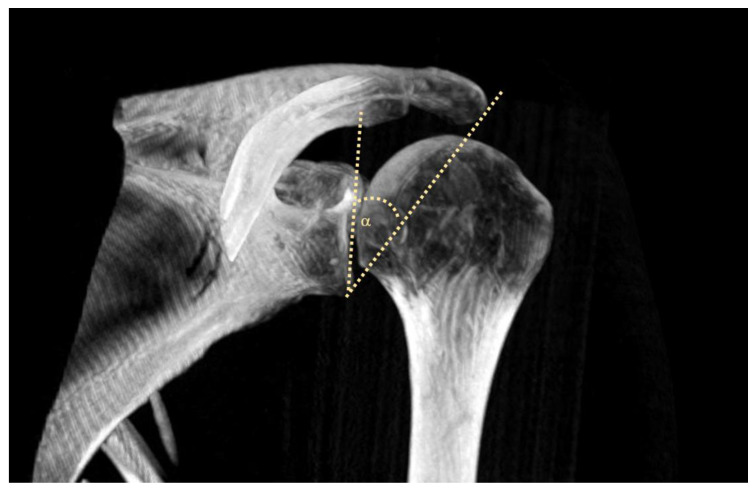
Three-dimensional reconstruction of a CT scan of the shoulder joint with drawn CSA.

The bony structure of the acromion represents a well-established risk factor that significantly affects the development of the outlet impingement and the likelihood of experiencing rotator cuff tears [1,2,3]. Furthermore, the relationship between the acromion, glenoid and humerus, known as the acromio-glenohumeral geometry (AGHG), can also be a risk factor for pathological changes in the shoulder joint [4]. Numerous metrics are available for evaluating the acromio-glenohumeral geometry. In the sagittal plane, the di Bigliani classification defines the acromion shape, and the acromio-humeral distance is suggested as a potential risk factor for rotator cuff tears. In the coronal plane, reported risk factors include the acromion index, the lateral acromial angle, and the critical shoulder angle [5,6,7,8]. The critical shoulder angle (CSA), initially described by Moor et al., is one of the key measures that is routinely used in clinical practice for the quantitative assessment of the shoulder joint in plain radiographs and sectional imaging (Figure 1) [8]. Used in a coronal section, the CSA is defined as the angle between the line connecting the upper and lower borders of the glenoid and the line from the lower edge of the glenoid to the lateral margin of the acromion; the CSA is defined by pathological thresholds below 30° and above 35° [8].

In a biomechanical investigation, Viehöfer et al. emphasized the effects of an altered CSA on shoulder abduction and showed that it increased the shear forces within the shoulder joint, especially effecting the subacromial space, including exposed parts of the rotator cuff [9]. Further investigations have revealed a significant correlation between a reduced CSA and the development of glenohumeral osteoarthritis [10]. Interestingly, in addition to the increased prevalence of glenohumeral osteoarthritis associated with a low CSA (<30°), an association has been identified with the pathologies of the rotator cuff in cases where the CSA exceeds 35°. Essentially, an abnormal CSA proves to be a recognizable risk factor for pathologies of the shoulder joint.

In recent years, in addition to the primary risk factors for the development of a rotator cuff rupture due to an altered acromio-glenohumeral geometry, the re-rupture rate after surgical rotator cuff repair, which is influenced by the above-mentioned geometric change, has also been discussed. A seminal work by Tauber et al. in 2020 demonstrated that correcting a pathological CSA (>35°) significantly reduces the risk of re-rupture following an arthroscopic intervention for rotator cuff ruptures [11]. Usually, CSA determination relies on plain radiographic X-rays, magnetic resonance imaging (MRI), or computed tomography (CT) [8,12,13,14]. Despite the well-defined bony landmarks characterizing CSA, a notable gap exists as there is currently no established method for sonographically guided CSA determination. In every imaging method, there are limitations to the visualization of structures. The most important limit in sonographic imaging is the fact that bony structures do not allow the underlying bony parts, as well as other anatomical regions, to be visualized. Due to the acromion being a physical obstacle, the glenoid, as a landmark, cannot be visualized by ultrasound, so other options had to be developed for the determination of the CSA using ultrasound.

This study attempts to investigate the reliability and validity of a modified CSA (mCSA) detectable by ultrasound. A comparative analysis with CSA determination via MRI has been undertaken, aiming to contribute insights into the potential of sonographically guided CSA assessments in clinical applications.

## 2. Methodology

### 2.1. Study Design and Patient Recruitment

This retrospective study was performed in accordance with the “Strengthening the Reporting of Observational Studies in Epidemiology (STROBE) Statement: guidelines for reporting observational studies” [15].

In this study, we enrolled patients presenting complaints related to the shoulder joint, where both magnetic resonance imaging (MRI) and sonographic examinations of the same shoulder joint were conducted between November 2021 and October 2022. The aim was to comprehensively assess and analyze archived MRI findings alongside their corresponding ultrasound evaluations.

### 2.2. Exclusion and Inclusion Criteria

Exclusion criteria were carefully established, including evident indications of advanced glenohumeral osteoarthritis (Kellgren–Lawrence grade of > °II) [16], instances of humeral head necrosis, the presence of open epiphyseal plates, recent or consolidated fractures involving the humeral head or glenoid, acromioclavicular joint disruptions, a history of shoulder joint total endoprosthesis implantation, and technically inadequate MRI or ultrasound examinations.

The MRI examinations of the shoulder joints were conducted using standardized coronal, sagittal, and axial planes with T1 or TSE weighting. The critical parameter CSA was precisely determined within the coronal plane.

Inclusion criteria were meticulously defined to ensure the validity of the study. They determined that a valid MRI examination in T1 and/or TSE weighting in the coronal plane should be available and that a sonographic examination of the same shoulder joint in the superior longitudinal section should be performed timely in accordance with the guidelines of the German Society for Ultrasound in Medicine (DEGUM) [17]. The additional inclusion criteria included being of legal age and having the capacity to provide consent for the study. Furthermore, only patients experiencing complaints in the shoulder joint area were included in the study.

### 2.3. Ultrasound-Assisted Assessment of the mCSA

For the sonographic examinations, a linear transducer with a variable ultrasound frequency ranging from 7 to 15 MHz was employed.

**Figure 2:**
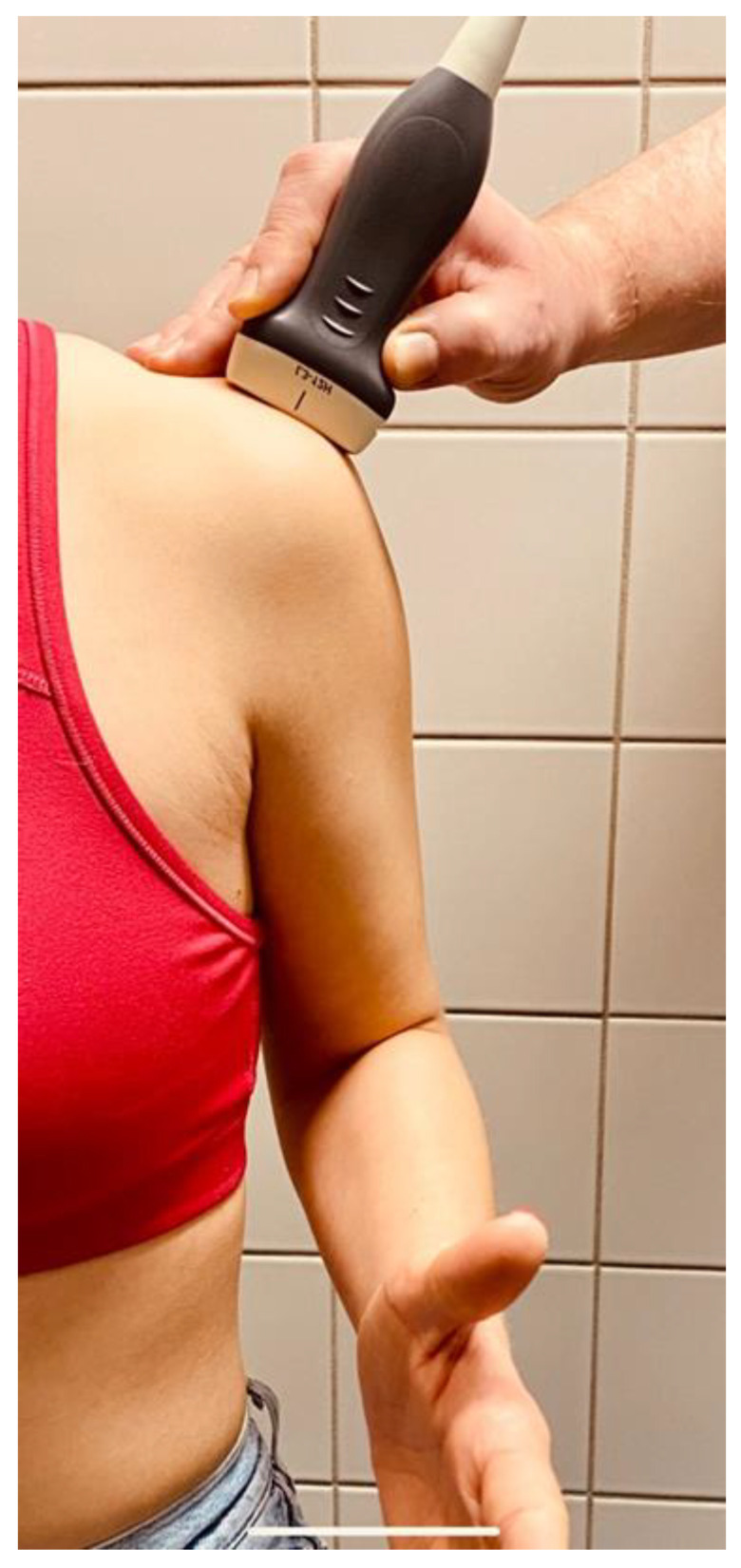
Positions of patient, arm, and transducer in sitting position.

**Figure 3:**
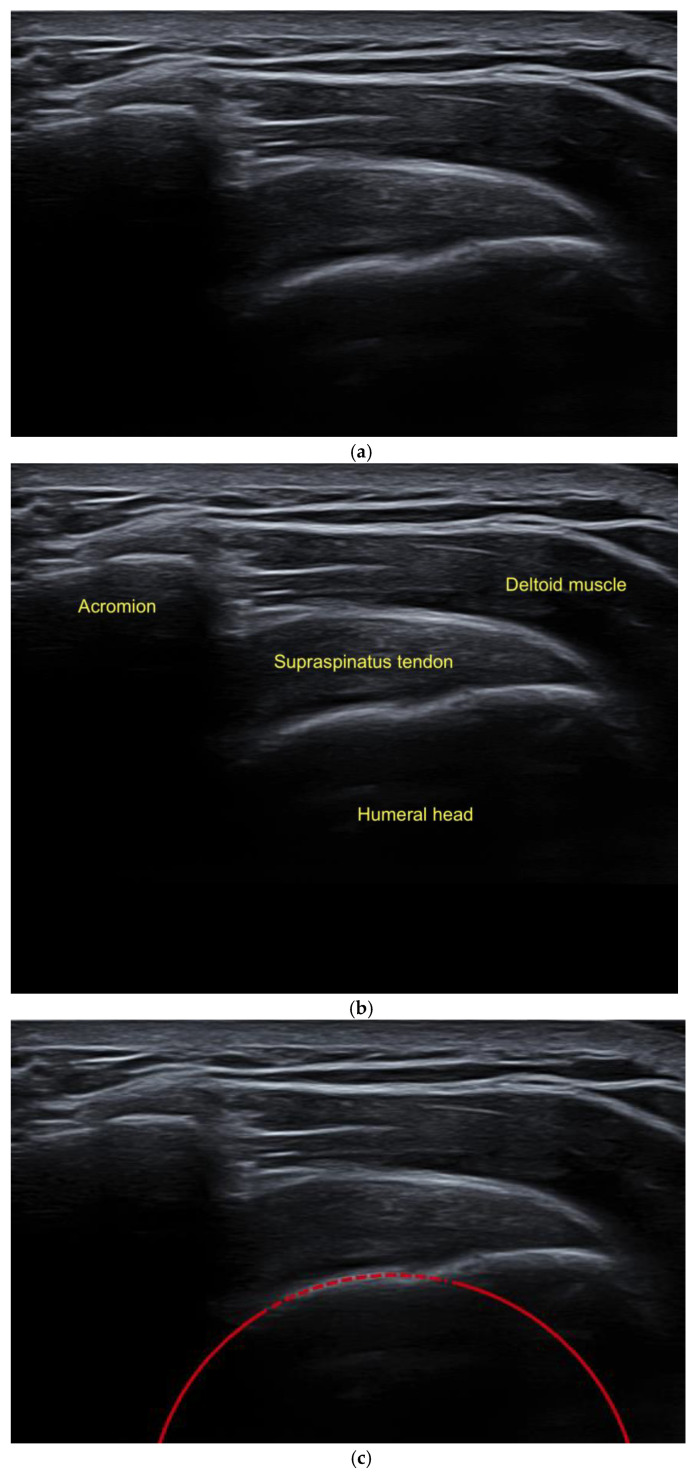

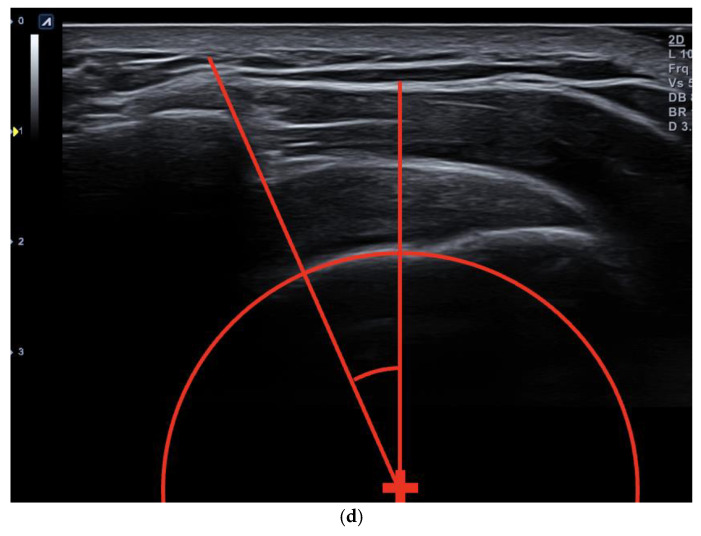
(**a**) Native ultrasound image of the shoulder joint in superior lateral section based on DEGUM guidelines. (**b**) Identification of the anatomical structures. (**c**) Expansion of the depicted circular section of the humeral head. (**d**) Determination of the mCSA.

1. The examination of a patient was performed in a sitting position, ensuring that the arm being examined was not placed in an abducted posture, and the circular structure of the humeral head was included in the imaging (Figure 2).
2. To ensure the accurate determination of the modified critical shoulder angle (mCSA), the lateral aspect of the acromion and the sphericity of the humeral head were visualized in the superior longitudinal section, and all relevant anatomical structures needed to be identified (Figure 3a,b).
3. Subsequently, utilizing an image viewing, editing, and measuring program (Centricity, Universal Viewer Zero Footprint Client, Version: 6.0 SP11.2.3), the sonographically visualized circular section of the humeral head was expanded to a complete circle. This method facilitated the precise identification of the central point of the humeral head (Figure 3c).
4. Following this, a systematic approach was employed to position the first arm vertically up to the identified center of the humeral head and perpendicular to the horizontal borders of the monitor (Figure 3d).
5. The second arm, originating from the circle’s center, was extended to the most lateral edge of the visualized acromion (Figure 3d).

### 2.4. MRI-Assisted Assessment of the CSA

**Figure 4:**
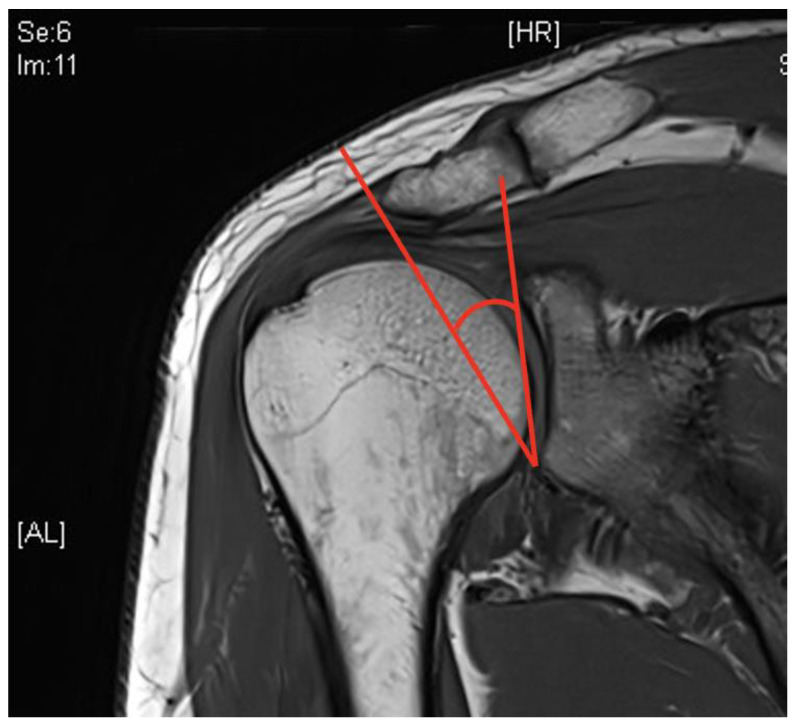
CSA depicted in an MRI in coronary sections (T1, 3 Tesla).

The MRI-based determination of the CSA was carried out with native MRI; however, the coronal planes were used for quantitative assessment in the sense of the CSA, involving the positioning of one arm of the angle as a connecting line between the superior and inferior borders of the glenoid fossa. The second arm was oriented from the lower edge of the glenoid fossa to the most lateral aspect of the acromion (Figure 4) [12].

### 2.5. Statistical Analysis

To ensure the reliability and objectivity of the study, the evaluation of both MRI and ultrasound images was undertaken independently by two certified examiners, following the guidelines set by DEGUM. Each examiner performed three measurements on ultrasound images and three measurements on MRI images so that a total of twelve measurements were performed for each joint over a specific period of time. This approach not only ensured precision in the analysis but also provided a robust basis for drawing meaningful conclusions regarding the CSA and the modifications in the shoulder joints under investigation.

For the statistical assessment, we employed SPSS software, version 27 (IBM SPSS Statistics for iOS, Armonk, NY, USA). The significance level was set at *p* ≤ 0.05. To achieve a robust effect size of η^2^ = 0.14 with a power of 0.9, a priori, a total of 103 participants were calculated for a meaningful outcome, and α was set to 0.05 [18]. The results of both methods were visualized and examined for correlation using linear regression and Pearson correlation, with a one-factor-ANOVA analysis employed to explore statistical differences. Conforming to Cohen’s guidelines, an a priori power analysis was conducted, establishing a sample size of *n* = 40 for a significant overall model, given a determination coefficient of R^2^ = 0.26 (indicating a large effect), a statistical power of 0.9, and a significance level of α = 0.05. For a sample size of *n* = 112, a statistical power of 0.9, and a significance level of α = 0.05, a determination coefficient of R^2^ = 0.104 was necessary for a significant overall model [18]. Interrater reliability was assessed, with a value of 0.7 or higher being considered satisfactory [19].

## 3. Results

### 3.1. Demography

A total of 112 patients (37/33% left and 75/67% right shoulder joints), with an average age of 54.7 years (range: 18–81 years; 40/35.7% female and 72/64.3% male), met the inclusion criteria and were enrolled in the study (Table 1). Three minor outliers were identified during the data analysis and excluded from the subsequent statistical analyses; thus, 109 patients underwent an MRI-assisted assessment of the CSA and an ultrasound-assisted assessment of the mCSA.

### 3.2. MRI-Assisted Assessment of the CSA

In the MRI scans, 53 patients (48.6%) exhibited a pathological CSA, with 34 patients (31.2%) having a CSA below 30° and 19 patients (17.4%) having a CSA above 35°. A total of 56 patients (51.3%) showed a normal CSA in the MRI. The gender distribution for the cohort with a CSA under 30° comprised 26 males (76.5%) and 8 females (23.5%), while the cohort with a CSA above 35° consisted of 15 males (78.9%) and 4 females (21.1%). The mean CSA determined in the MRI was 31.5° (range: 24.8–41.5°; SD: 3.899) (Table 2 and Table 3).

### 3.3. Ultrasound-Assisted Assessment of mCSA

Following linear regression, in line with Moor et al.’s guidelines, an mCSA of 28.2° in ultrasound for an MRI CSA of 30° and an mCSA of 31.8° for an MRI CSA of 35° were determined [8].

In summary, it must be noted that a high mCSA in ultrasound equals a low CSA in MRI, while a low mCSA equals a high CSA in MRI. In the ultrasound examination, 44 patients (40.4%) had an mCSA below 28.2°, and 39 patients (35.8%) had an mCSA above 31.8°, indicating a total of 83 patients (76.1%) with a pathologically altered mCSA, and 26 patients (23.9%) showed a normal mCSA. The gender distribution for the cohort with an mCSA under 28.2° comprised 28 males (63.6%) and 16 females (36.4%), while the cohort with an mCSA above 31.8° included 26 males (66.7%) and 13 females (33.3%). The mean mCSA determined in ultrasound was 30.1° (range: 20.3–39.9°; SD: 4.7529). High interrater reliability was observed for the determination of both the CSA (0.993) and mCSA (0.989). The intrarater reliability was factual with 0.967 for CSA and 0.948 for mCSA (Table 2 and Table 3).

### 3.4. Comparative Analysis

**Figure 5:**
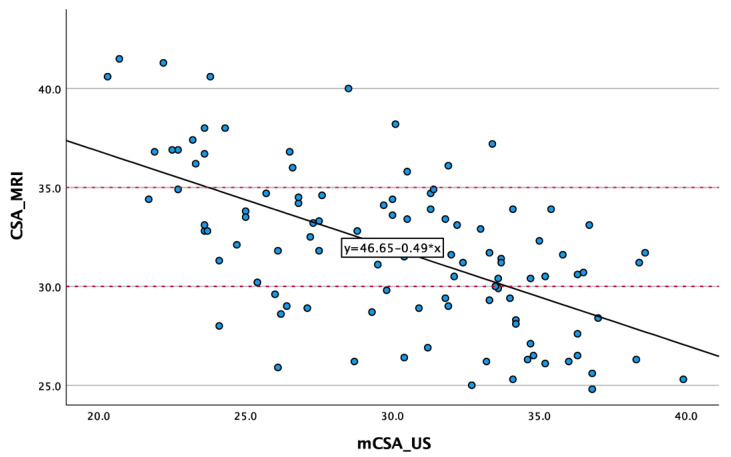
Scatter plot between MRI and ultrasound.

Both the CSA (MRI) and the mCSA (ultrasound) exhibited normal distributions based on the Shapiro–Wilk test (*p* > 0.05). The linearity and linear regression were examined, revealing an R^2^ of 0.358 (Figure 5). An ANOVA indicated a substantial effect size of η^2^ = 0.358. The CSA determined in the MRI displayed a strong correlation with the mCSA determined in ultrasound (r = −0.598; *p* < 0.001).

## 4. Discussion

The results of the present study demonstrate, for the first time, that the sonographically guided determination of the modified CSA is a safe, reproducible, and valid method compared to the MRI-based determination of the standard CSA. A significant difference was not found between the two examination methods.

According to the German guidelines for the rotator cuff, an ultrasound diagnosis is listed as an integral part of the diagnostic algorithm within advanced imaging methods. In this study, a new diagnostic parameter was introduced, which, in addition to an ultrasound examination, does not require further imaging and can be used equivalently to the already established and generally accepted CSA in clinical practice. This allows for the supplementation of the examination with a quantitative parameter, such as the mCSA (modified critical shoulder angle), alongside the direct assessment of the rotator cuff in terms of qualitative evaluation [20].

In this study, the provided data estimated a mean CSA of 31.5° in MRI and a mean of 30.1° for mCSA in ultrasound, respectively, which are comparable values with those in the current literature [13,21].

The determination of the CSA represents an established procedure for the quantitative assessment of the acromio-glenohumeral geometry. Moor et al. examined the clinical relevance with a CSA altered in planar radiographic imaging and established normal values between 30 and 35° [8]. There was a high correlation between these values and the ultrasound-estimated mCSA that was newly introduced in this study.

The meta-analysis published in 2020 by Smith et al. highlighted the quality of X-ray images as a significant influencing factor for the accurate determination of the CSA [22].

In everyday clinical practice, it is not always possible to obtain a correct, true antero-posterior X-ray image of the shoulder, which can have a negative effect on the measurement of the CSA, as the specified measurement points on the glenoid cannot be determined correctly, and the anatomically most lateral point of the acromion does not appear correctly at the corresponding point on the X-ray. In addition to conventional X-rays, using CT as an imaging technique is a valid method for determining the CSA, although the radiation exposure for the patient must again be mentioned as a disadvantage of this method. Previously, MRI was the only available imaging procedure to determine the critical shoulder angle without exposing the patient to radiation. In the present study, however, it is shown that the ultrasound-guided determination of the CSA could be used effectively as a further radiation-free imaging procedure.

Biomechanical studies, such as those by Viehöfer et al., have demonstrated that changes in the CSA can have significant effects on shoulder abduction and can increase shear forces in the shoulder joint [9].

Furthermore, extensive research has established a connection between a reduced CSA and the development of osteoarthrosis in the shoulder joint [10]. Abnormal CSA values have not only been linked to an increased prevalence of osteoarthrosis in the shoulder joint, but also to pathologies affecting the rotator cuff. Essentially, abnormal CSA values emerge as significant risk factors predisposing the shoulder joint to pathological conditions. A groundbreaking study by Tauber et al. in 2020 showed that correcting a pathological CSA significantly reduces the risk of re-rupture following the arthroscopic repair of rotator cuff tears, which underlines the usage and the need for the evaluation of the bony structures of the shoulder joint via CSA, especially after rotator cuff repair [11]. To spare ionized radiation, X-rays should be reduced to a minimum, and an alternative examination method should be established. As a new imaging method for assessing the acromio-glenohumeral geometry, ultrasound could also be used intraoperatively in the future as a radiation-free alternative to x-rays for the assessment of the sufficiency of a lateral acromioplasty.

In their double-blinded randomized study in 2021, Garcia et al. found that MRI and planar radiographic X-rays have high correlations with CSA determination [21]. In our study, an average CSA of 31.5° was determined, which only slightly differs from the values found in previous studies [21]. Since the mCSA is a newly introduced angle for assessing the shoulder morphology, there are currently no comparable studies. It is remarkable that, compared to the conventional CSA with high correlation, no diagnostic loss of quality is observed.

The radiation-free nature is a particular advantage of the sonographically guided method. Additionally, it is a cost-effective, widely available, and rapidly accessible examination method. As an established diagnostic tool for evaluating the shoulder joint, ultrasound examination can provide reliable information about possible changes, especially in the soft tissues surrounding the shoulder, such as the rotator cuff, potentially eliminating the need for further diagnostic procedures. Unlike conventional X-rays, which require adequate positioning for CSA determination, ultrasound imaging allows for a continuous, radiation-free improvement in the image until an optimal setting for mCSA determination is found. In typical sectional imaging methods, such as MRI and CT, the maximum expression of the acromion may lie between the slices, which are usually 2–4 mm thick [23]. This source of error can be eliminated in ultrasound, as it is a dynamic examination with live imaging [17].

Subcortical changes cannot be assessed through ultrasound examination. Furthermore, the severe destruction of the humeral head, which can occur in advanced arthritic changes, poses a challenge for the described technique, as a circular section may not be defined, and thus, the center of the humeral head cannot be defined. Additionally, during data analysis, it was found that an optimal arm position, as defined for the standard cuts of the DEGUM, must be maintained [17,24,25]. For example, if the upper arm is positioned in abduction, a circular section cannot be defined because the greater tubercle is then located in the displayed image, and the circular section can no longer be expanded to a full circle.

This study has some limitations: Firstly, its retrospective study design should be mentioned. A randomized study design could potentially yield different results. Due to the newly described technique, the thresholds for a normal mCSA (28.8–31.8°) must be graphically determined. Further investigations are needed to narrow down the reference values and compare them to pathologies such as rotator cuff changes or osteoarthritis. The currently established values represent a very small range of 3°, which could limit this method’s practicality in clinical practice due to measurement inaccuracies. In the statistical analysis, a discrepancy was observed between the MRI and ultrasound values in the range of >35° in MRI and under 28.2° in ultrasound. An explanation for this might be the reduced visibility of the humeral head segment, which is needed for the completion of the circular section, due to increased coverage by the acromion.

An improved resolution; new technical applications, such as the ultrasound-assisted 3D imaging of joints; as well as an increased image quality could further enhance the relevance of the sonographic assessment of the mCSA in the coming years.

In their study, Qi et al. were able to show that the use of a combination of predictors is better suited for predicting rotator cuff tears than the use of a single parameter alone. Therefore, further sonographically determinable predictors should be identified in subsequent studies in order to achieve the advantages of radiation-free examination with the higher accuracy of combining several measurement methods to predict damage in the rotator cuff [26].

## 5. Summary

The evaluation of the mCSA using ultrasound examination is a secure and valid method. Therefore, the present study established a high correlation between a sonographically determined mCSA and a tomographically determined CSA using MRI. No significant differences were observed between MRI and sonography. Despite this study’s retrospective, monocentric design that only evaluated Caucasians/Central Europeans, and considering the known limitations of sonographic imaging, it was demonstrated that sonography can be utilized as a simple, cost-effective, rapid, and radiation-free technique for determining the mCSA. This method showed a very good correlation with the standard CSA without any loss of diagnostic quality.

## Figures and Tables

**Table 1:** Demography.

Side		Sex		Age
left	right	male	female	mean
37 (33%)	75 (67%)	72 (64.3%)	40 (35.7%)	54.7 years

**Table 2:** Average values of CSA/mCSA.

	CSA in MRI	mCSA in US
**Mean**	31.5°	30.1°
**Range**	24.8–41.5°	20.3–39.9°
**Standard Deviation**	3.899°	4.752°

**Table 3:** Distribution of pathological CSA in MRI and mCSA in ultrasound.

Pathological CSA in MRI		Pathological mCSA in Ultrasound	
<30°	>35°	<28.2°	>31.8°
34 (31.2%)	19 (17.4%)	44 (40.4%)	39 (35.8%)

## Data Availability

The data presented in this study are available on request from the corresponding author.
